# Reflectance spectroscopy: a non-invasive strategy to explore skin reactions to topical products

**DOI:** 10.3389/fchem.2024.1422616

**Published:** 2024-06-18

**Authors:** Antonia Mancuso, Nicola d’Avanzo, Maria Chiara Cristiano, Donatella Paolino

**Affiliations:** ^1^ Department of Experimental and Clinical Medicine, “Magna Graecia” University of Catanzaro, Catanzaro, Italy; ^2^ Research Center “ProHealth Translational Hub”, “Magna Graecia” University of Catanzaro, Catanzaro, Italy; ^3^ Department of Medical and Surgical Sciences, “Magna Graecia” University of Catanzaro, Catanzaro, Italy

**Keywords:** reflectance spectroscopy, skin, analytical technique, refraction, diffusion

## Abstract

Reflectance spectroscopy has emerged as a powerful analytical technique in the field of dermatology, offering a non-invasive strategy to assess several cutaneous properties and skin response to topical products. By analyzing reflected light across different wavelengths, reflectance spectroscopy allows the quantification of cutaneous parameters, such as erythema index and melanin content. Moreover, this analytical technique enables the monitoring of any changes in skin physiology facilitating the assessment of long-term effects of topical products as well as predicting cutaneous diseases. This review provides an overview of the application of reflectance spectroscopy in investigating skin properties and reaction to topical applied products, including both pharmaceutical and cosmetic formulations, thereby aiding in the development of personalized solutions tailored to individual needs.

## 1 Introduction

It is well known that the skin is the largest organ of the human body (Supe and Takudage, 2021) and that it acts as a protective barrier against physical, chemical, and biological factors also playing a crucial role in sensory perception (Harris-Tryon and Grice, 2022). Considering its functions, the human skin is highly dynamic and constantly responds to various stimuli, that are intrinsic and physiological (such as ageing) or extrinsic (such as environmental changes or exposure to UV radiations and/or different substances, including pharmaceutical and cosmetical products) (Bang and Choung, 2020; Blair et al., 2020). Each stimulus can be responsible for specific skin’s reactions, which are helpful in the evaluation of drug safety profiles, their pharmacological activity as well as the effects of products applied on the epidermis with cosmeceuticals purposes.

In fact, despite the difficulty of crossing the skin barrier (Yu et al., 2021), the topical application of drugs is preferred over other routes of administration both to exert local efficacy and (in some cases) to also exhibits a systemic action (de Macedo et al., 2020; Tijani et al., 2021; Pathania et al., 2022), stimulating growing scientific research for the design of new topical pharmaceutical formulations. For both pharmaceutical and cosmetic products, the application on skin represents a non-invasive, comfortable, and appreciated administration route, preferred by children and the older patients, particularly when involved in chronic therapies or treatments.

When a new formulation is tested, the assessment of skin reactions to topical products deeply relies on biased evaluations, such as visual inspection and patient- or customer-reported outcomes. While these methods provide valuable information, they often lack precision, reliability, and objectivity. On the other side, although greatly informative, the more invasive techniques such as skin biopsies are not practical for routine evaluation due to their discomfort and potential risks, including irritation, damage, or alteration of skin tissue (Logger et al., 2020).

Among the non-invasive techniques applied to human skin, reflectance spectroscopy has emerged as a promising technique for facilitating a comprehensive assessment of skin pathophysiology and responses to therapeutic reactions of topical products (Chen et al., 2023). By analyzing the interaction of the incident light with the skin, reflectance spectroscopy offers a wide range of data regarding skin physiology, morphology, and biochemical composition, monitoring changes in skin parameters before and after product application, thereby elucidating their effects on barrier function, pigmentation, and other relevant metrics.

Thanks to the possibility to record several parameters, in recent years, reflectance spectroscopy has become popular in various fields, including dermatology, cosmetics, and pharmaceuticals, due to its versatility, accuracy, and non-invasiveness. This technique encompasses a range of modalities such as diffuse reflectance spectroscopy, hyperspectral imaging, and Raman spectroscopy, each offering unique insights into skin properties and reactions.

This review aims to provide a recent literature revision on reflectance spectroscopy as a non-invasive strategy for exploring skin reactions to topical products. Hence, we discuss the principles inherent in the reflectance spectroscopy and explore the applications of this technique in dermatological research and cosmetic formulation. Furthermore, we critically highlight the advantages and limitations of this technique compared to traditional methods, providing a better understanding of its potential to revolutionize the assessment of skin responses to topical interventions.

## 2 Reflectance spectroscopy

Reflectance spectroscopy represents a powerful analytical technique that has different implications in several scientific fields, including material science, chemistry, and biomedical applications (Morgan et al., 2016; Nilsson et al., 2017; Vananti et al., 2017; Mishra et al., 2018). This technique focuses on the measurement of the intensity of light reflected from a generic sample across different wavelengths (Wallace et al., 2009). By studying the reflected light, several information regarding the composition of the sample, the structure and other properties can be collected (Frei, 2019). Indeed, reflectance spectroscopy relies on the principle that different materials interact with light in unique ways, leading to distinctive spectral signatures (Ball, 2001). These signatures can be exploited for quantitative and qualitative analysis identifying any substance in a mixture and determining the concentration of substances, respectively (Ball, 2001; Frei, 2019). Furthermore, reflectance spectroscopy can provide insight into chemical interactions, electronic properties of materials and their surface morphology as well as distinguish between normal and diseased tissues (De Boer et al., 2018).

The reflectance spectrum is normally obtained using a spectrophotometer which compares the intensity of light incident to a generic sample and that reflected from it (Yoon and Yoon, 2016).

Several measurements such as diffuse reflectance, specular reflectance and hemispherical reflectance offer insights into various material properties (Nichelatti et al., 2007). Diffuse reflectance spectroscopy provides measurements of the scattered light from a sample surface; specular reflectance focuses on the reflection of light at specific angles from a smooth surface providing information about the roughness of a thin surface; hemispherical reflectance measures the total reflected light from a sample surface over a hemisphere and is particularly useful for curved surfaces. Here we focused on diffuse reflectance, which can be measured through spectroscopic or imaging methods and is particularly useful in the field of biomedical applications (Moy and Tunnell, 2016).

As an example, the skin can be subjected to optical diagnostic methods including diffuse reflectance spectroscopy (DRS) and diffuse reflectance imaging (DRI) being easily accessible. When an incident radiation encounters the skin, part of it is absorbed while the rest is partially reflected. Indeed, the photons are subjected to various interactions with the skin components (such as blood vessels, extracellular matrix, and other elements), which deviate their path and cause a partial scattering of light after a random number of events (Moy and Tunnell, 2016). Scattering coefficient, absorption coefficient and anisotropy define the interaction between light and skin, considering anisotropy as the quantification of forward scattering occurring in the tissue (Soltaninezhad et al., 2020).

Following the interaction with skin, as well as other biological tissues, the remitting light can be monitored in the diffuse reflectance, which is calculated in terms of light absorbed and scattered, and finally used to investigate the amounts of skin molecular components, i.e., skin chromophores. The principal chromophores of skin that guide the optical absorption are melanin, haemoglobin, carotene, and bilirubin, which have a specific range of values in healthy skin and can be used to predict suspicious lesions in case of significant alterations (Elisabeth, 2020).

From a clinical point of view, skin lesions cause alterations in optical properties leading to a darkening of skin and suggesting increased absorption, which can be detected using reflectance spectroscopy (Moy and Tunnell, 2016). Other diseases, such as basal cell carcinoma, lead to alterations in collagen and cell organization with a consequent decrease in optical light/photons scattering, which can be predictive of alterations, typical of a non-healthy skin (Rajaram et al., 2010; Moy and Tunnell, 2016).

Measurement of DRS is a rapid and non-invasive way to detect any cutaneous alteration. The wavelength is calculated according to the following equation (Moy and Tunnell, 2016):

$$\text{DRS }\left(\lambda \right)=\frac{I_{skin}-I_{background}}{I_{standard}-I_{background}}$$

Where I_skin_ is the reflected light intensity spectra obtained from the skin, I_background_ is the background intensity spectra, I_standard_ is the reflected light intensity spectra recorded from a well-known reflectance standard.

Following the collection of data relative to diffuse reflected light, the spectrophotometer will finally provide spectral analyses. This tool is very simple to use and is composed of a probe, which is directly placed on the sample or skin to emit light and collect spectra. The spectrophotometer is able to measure the entire spectral composition of light (360–700 nm) (Morris, 2008). It always provides systems for the distribution of a wide range of illuminants, and then it highlights color differences that are not visible to the naked eye. Halogen lamps, xenon lamps and white Light Emitting Diodes (LEDs) are only some examples of light sources for DRS (Moy and Tunnell, 2016).

Similarly to DRS, DRI represents a quantitative technique though it determines optical absorption and optical scattering through images of diffuse reflectance.

Information about pigmentation and erythema as well as cutaneous physiology can be easily evaluated by calculating the intensity of light reflected from skin. Regarding these evaluations, it is important to mention the CIE color system, which is an international standard adopted by the Commission Internationale d'Eclairage (CIE) which gives an objective color estimation (Green, 2023). One of the most widely used CIE color system is CIE L*a*b* which operates considering L*, a* and b* values referred to lightness, red/green intensity, and yellow/blue intensity, respectively. Each value correlates with cutaneous parameters, where a* parameter is useful to quantify erythema, while L* and b* indicate the degree of pigmentation (Fajuyigbe et al., 2018; Das et al., 2021). More specifically, L* indicates lightness, measuring the intensity of pigmentation on a scale from 0 (black) to 100 (white). The a* coordinate indicates the green-red axis, ranging from −127 to +127, reflecting skin redness—higher positive values indicate greater redness or inflammation. Meanwhile, b* signifies the blue-yellow axis within the same range, correlating with the yellowness or sallowness of the skin. Using this system, it is possible to compare distinct color spaces excluding variations dependent on devices or visual skills of the observer.

Commonly, a generic colorimeter breaks the light down into three primary colors (red, green, and blue–RGB components) similar to the human eye. However, color’s numeric values are subsequently obtained using CIE color space, interpreting measurements in a color space graph. Also, colorimetric devices present great potential in clinical application detecting skin colors using the L*a*b* values. Nevertheless they are limited by their inability to distinguish between metameric colors, characterized by superimposable appearance but with spectral divergences.

**FIGURE 1:**
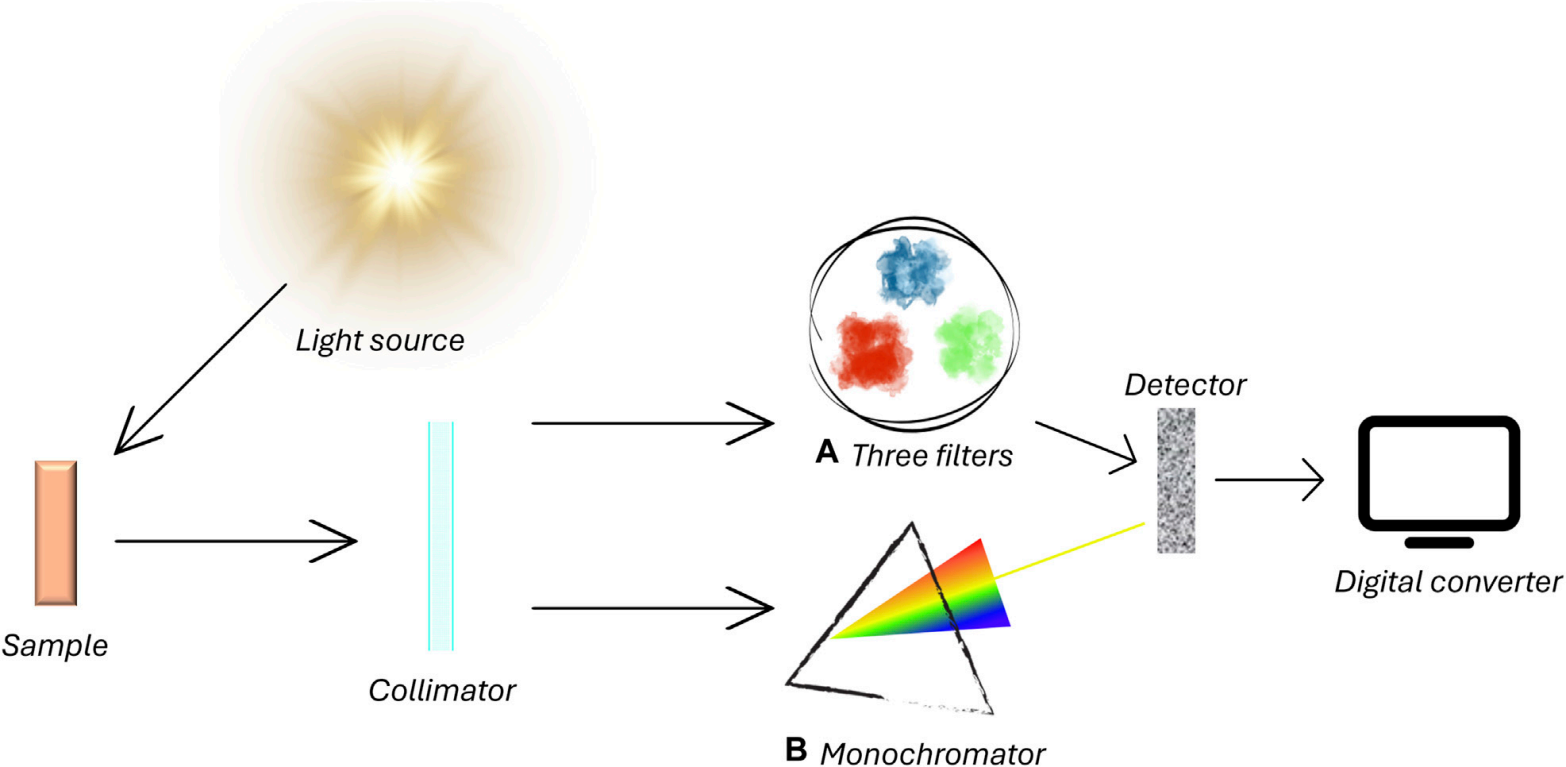
Operating principles of a) colorimeter and b) spectrophotometer. The general operating principles are similar between both systems. A light source emits wavelength onto a sample. Reflected light is captured by a colorimeter and filtered through the trichromatic filter or goes through a device that selects wavelengths between 360 and 700 nm. Specific spectral sensors process data sending them to a digital converter.

Overall, colorimeters and spectrophotometers are mostly used for the quantification of colors and both often involve the analysis of the wavelength that is reflected from the sample (Figure 1).

## 3 Topical application of reflectance spectroscopy

In the last decades, the potential use of spectroscopy in clinical and biomedical fields paved the way to an improvement among skin instrumental assessment. Despite the main exponents of spectrophotometers being designed for operating across the entire optical spectrum (from ultraviolet to near-infrared part), the majority of scientific evidence reported in recent literature has been based on the use of reflectance spectroscopy in the visible spectrum, where is possible to find the absorption peaks of important cutaneous chromophores (Minakawa et al., 2023).

The major chromophores found within the outermost layer of skin comprise haemoglobin and melanin and possess unique optical absorption characteristics within the visible wavelength spectrum (Kikuchi et al., 2015). Haemoglobin can exist in the microvascular network of the dermis in two forms: oxygenated and deoxygenated. The absorption spectra of these haemoglobin derivatives exhibit noticeable distinctions (Nkengne et al., 2018), attributable to the alterations in light absorption spectrum brought about by the binding of oxygen to haemoglobin. Monitoring the amount of oxygenated haemoglobin in comparison with total haemoglobin in a tissue, the tissue oxygen saturation can be estimated and used as an indicator of peripheral tissue oxygen condition.

Melanin is a natural pigment of skin, located in the epidermis and it possesses several biological functions, above all protection from solar radiation and antioxidant defense (Solano, 2020). On the other hand, the production of melanin is involved in pigmented skin lesions such as freckles, lentigines, melasma (Thawabteh et al., 2023), seborrheic keratosis (Cheong et al., 2021), solar keratosis, basal cell carcinoma, and melanoma (Slominski et al., 2022).

The skin color is due to the combination of skin chromophores red (oxyhaemoglobin), blue (deoxygenated haemoglobin), and brown (melanin) (Ortonne, 2012). Fortunately, a variation in skin color due to chromophores concentration variation is not always related to severe pathological conditions but it can be related to skin response to some external stimuli, such as the application of topical products. Reflectance spectroscopy has been *in vivo* used by some research groups to monitor the safety profile of pharmaceutical and cosmetical products or the pharmacological efficacy of topical formulations containing, for example, anti-inflammatory drugs, but also for monitoring eventual UV-induced erythema (Liebold et al., 2000) (Table 1). This non-invasive technique can be useful during the design and characterization of new topical products since the skin could respond to new formulations or new raw materials (for example, preservatives) with undesirable signs (Bilal and Iqbal, 2019), such as irritation, redness, and skin color changes, often not visible to the naked eye. In particular, the redness possibly induced by cosmetics is taken as an indicator of inflammation, but it cannot be used alone to assess all types of skin reactions (Hermanns et al., 2000). Erythema is produced when irritants, allergens and short-wavelength ultraviolet light applied on the skin induce a dilation of blood vessels close to the skin surface. Skin color can be measured instrumentally using reflectance techniques, taking into account the quantification of haemoglobin (erythema index), and obtaining results more objective, reproducible, and quantitative data than visual scoring. The first reflectance spectrophotometers were initially developed for reproducible color documentation in paint and textile industry; it was later proposed to use broadband visible light to quantify erythema. This new approach is based on the tristimulus L*a*b* system of the CIE (ISO/CIE 11664-4:2019). Several studies have been performed from our research group using reflectance spectrophotometer, with the aim to evaluate the safety and the efficacy profiles of topical formulations, by measuring erythema index before and after application of products on human healthy volunteers. The method is ancient although poorly used for this specific purpose. This approach of reflectance analysis provides spectra of skin reflectance, typically within the 400–700 nm range. These spectra allow for the derivation of various color space values (such as CIELab, Lch, *etc.*) using different CIE illuminants (C, D65, D50, A, *etc.*) and illuminant observers at either 2 or 10°. By analyzing spectral data, it becomes possible to compute the relative reflectance or the logarithm of inverse reflectance (LIR) at different wavelengths, indicating the absorption of skin chromophores like haemoglobin and melanin. As erythema primarily results from increased haemoglobin content in skin vessels, EI values are determined by subtracting LIR values at 510 and 610 nm (mainly reflecting melanin absorption) from the sum of haemoglobin LIR values at 540, 560, and 580 nm, corresponding to haemoglobin absorption peaks (Saija et al., 2000). The following equation permits to calculate a possible induced erythema:

$$EI=100\left[\log\frac{1}{R560}+1.5\left(\log\frac{1}{R540}+\log\frac{1}{R580}\right)-2\left(\log\frac{1}{R510}+\log\frac{1}{R610}\right)\right]$$

where R symbolizes the reflectance at a specific wavelength (510, 540, 560, 580, 610).

**TABLE 1:** Application of reflectance spectroscopy in cosmetic, nutraceutical and pharmaceutical fields.

Category	Products/Key ingredients	Investigation	Reference
Cosmetics	Face cream (O/W emulsion) containing olive leaves and citrus peels extracts; nanoemulsions for vitamin E delivery	Skin tolerance as variations of erythema index values (ΔE.I.) compared to baseline values	d Avanzo et al. (2024), Rinaldi et al. (2022)
	Syndet, glycerin, and creamy soaps	Skin erythema appearance compared to traditional alkaline soaps	Khosrowpour et al. (2019)
	Multiple emulsions containing ferulic acid	Photoprotective Effects against UV-induced skin damage	Mancuso et al. (2021)
	Farmaka Rosacea Cream	Soothing and reepithelization properties	Sparavigna et al. (2014)
	Herbal cream	Reduction in erythema in sensitive skin	Zhang et al. (2021)
	Soaked cotton pads and cosmeceutical facial masks	Investigation of skin absorption and scattering modulation caused by changes in skin hydration state	Chen et al. (2023)
	Whitening agents	Detection of suspicious products	Deconinck et al. (2014)
	Reflective particles and functional groups	Non-destructive characterisation of lipsticks or nail polish for forensic purposes	Wong et al. (2019), Chophi et al. (2021)
Nutraceuticals	Orange extract supplementation	Photoprotective Effects against UV-induced skin damage	Puglia et al. (2014)
	Eicosapentaenoic acid	Evaluation of any difference in the slope of the dose response curve post supplementation with EPA compared to control group treated with placebo	Pilkington et al. (2014)
	Oral supplementation with peptides and chrysanthemum extracts	Depigmenting efficacy for melasma	Gui et al. (2017)
Pharmaceuticals	Ultradeformable nanocarriers containing ammonium glycyrrhizate; rutin-loaded nanovesicles; thymoquinone-loaded oleic acid-based vesicular nanocarriers containing thymoquinone; nanoemulsions	Anti-inflammatory effects on skin treated with methyl-nicotinate solution as irritative agent	Barone et al. (2020), Cristiano et al. (2021), Bruno et al. (2022), Rinaldi et al. (2022)
	Nanomedicines contained a combination of drugs and other active compounds	Anti-inflammatory effects on skin treated with methyl-nicotinate solution as irritative agent	Cristiano et al. (2022), d Avanzo et al. (2022)
	Salicylic acid with penetration enhancer	Non-invasive diagnosis of dermatoses and therapeutic monitoring in clinical dermatology	Zhao et al. (2016)

**FIGURE 2:**
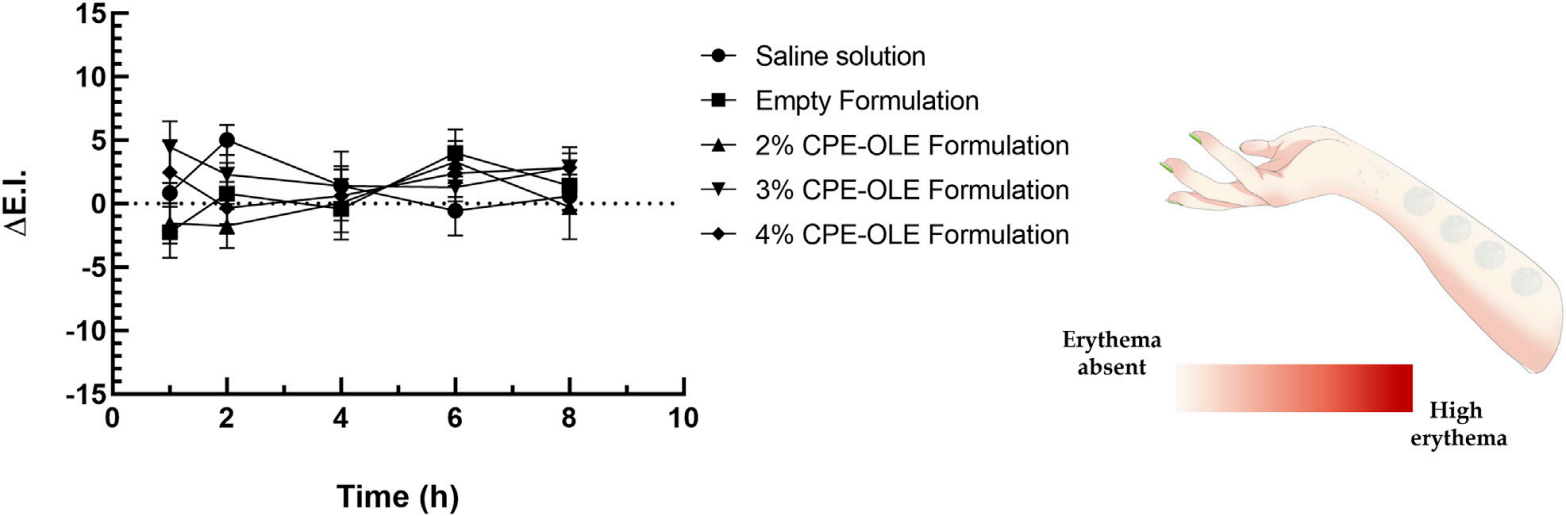
Variation of erythematous index values after the administration of cosmetic emulsions containing natural extracts. Saline solution (NaCl 0.9% w/v) was used as control studies. Reproduced with permission from (41), Cosmetics; published by MDPI, 2024.

In a very recent scientific work (d Avanzo et al., 2024), we used X-Rite Ci62 spectrophotometer to detect any variations of skin color following the application of cosmetic emulsions prepared by using natural extracts. In this work, baseline values of the erythema index (E.I.) were measured for each anatomical site considered in the study, and they were compared with the erythema index values re-obtained after prefixed times (Figure 2). We have demonstrated, by using these non-invasive and comfortable techniques, that the proposed cosmetic emulsions did not induce any detectable variation in erythema index compared to the baseline values, confirming the safety profile of samples and excluding irritating effects of tested products (d Avanzo et al., 2024). On the contrary, the proposed formulations have proven to be able to mask this effect, probably also thanks to the soothing action of the excipients used, such as sweet almond oil (Ramadan, 2019).

In the study mentioned, the forearms of human volunteers have been chosen as application sites for topical formulation. Certainly, forearms are considered comfortable sites for human tests, but care must be taken in choosing specific sites on the forearm. Generally, the most peripheral positions, close to the wrist and elbow, are particularly reactive than others (Sivesind and Maibach, 2022); moreover, often the two forearms of each human volunteer could induce the detection of different baseline values. For these reasons, it is very important to standardize the procedure of testing, to limit the analysis on the same longitudinal axis limited to the mid-forearm, and to increase the number of replicates.

Another work investigated the safety profile of four commercially available soap bars (alkaline soap bar, glycerin soap bar, creamy soap bar, and syndet bar) (Khosrowpour et al., 2019), considering that soaps and surfactants can cause irritant skin reactions (Van Scott and Lyon, 1953). In this work two parameters for stratum corneum integrity have been evaluated: transepidermal water loss and erythema index, indicating different potential damages affecting the skin. The results obtained in the study of Khosrowpour and co-authors highlighted a marked difference in skin reactions after the application of the four soap bars. In detail, the erythema index was monitored by using a reflectance spectrophotometer and it has been demonstrated that the erythema index was reduced significantly 72 h after the application of the creamy soap sample, and the author assumed that this reduction was due to the presence of lanolin in the tested formulation (Wright et al., 2016). This reduction in the erythema index and therefore the restoration of the basal condition of the skin were not recorded in the skin sites where alkaline soap was tested.

A similar experimental design is followed by several research groups to evaluate not only the skin tolerability of formulation but also the efficacy of cosmetic/pharmaceutical topical formulation in reducing erythema, redness, and other skin signs (Puglia et al., 2014; Sparavigna et al., 2014; Cristiano et al., 2021; Mancuso et al., 2021; Zhang et al., 2021; Cristiano et al., 2022).

For monitoring the efficacy of topical formulations or nutraceutical supplements on skin health and on the resolution of skin erythema, two paths can be pursued: testing the formulation on human volunteers already affected by skin signs or testing the formulation on human healthy volunteers after having deliberately induced skin erythema using a chemical agent or UV lamp (Puglia et al., 2014; Sparavigna et al., 2014; Barone et al., 2020; Zhang et al., 2021; Cristiano et al., 2022).

Recruiting human volunteers suffering from a particular skin condition is not simple but very useful for evaluating the effectiveness and tolerability of a certain formulation. This is the case of the scientific work carried out by Sparavigna et al. (2014), in which the use of reflectance spectroscopy was essential to demonstrate the efficacy of a topical formulation for rosacea. Since rosacea is a chronic inflammation skin disorder characterized by persistent redness and appearance of visible blood vessels (Two et al., 2015), reflectance spectroscopy is a suitable method for monitoring the progression/regression of the pathology (Logger et al., 2020). The formulation tested by the authors was a topical formulation prepared by using a patented combination of ingredients that promised moisturizing, restoring and hydrating effects on the skin. During the long-term study, the human volunteers affected by rosacea were enrolled and invited to apply the tested formulation for 8 weeks. The erythema index evaluation, by using non-invasive reflectance spectroscopy, was performed on the face at time 0 (used as baseline, pretreatment), after 4 weeks of treatments (as intermediate time) and at the final Week 8 visit. This technique enabled the identification of very small variations in erythema index, which are indicative signs of rosacea but are not appreciable to the naked eye. The authors of this study demonstrated that the proposed formulation was able to induce the reduction of rosacea signs (erythema and haemoglobin index) already after 4 weeks of treatments.

Another example of reflectance spectroscopy application for confirming the efficacy of topical formulation has been presented by Zhang et al. (2021). Also in this case, the study was carried out on human volunteers already affected by skin disorders; in detail 35 human female volunteers with visible and persistent erythema on the face upon exposure to ordinary skin care products were enrolled and were invited to apply 2 pumps of one of two creams (control cream and natural extracts-loaded cream) to each side of the face twice daily. After a treatment period of 28 days, the skin application of both formulations induced an improvement of skin conditions in volunteers, recording a significant reduction in a* values, analyzed by reflectance spectrophotometer, indicating a reduction in erythema index (Fajuyigbe et al., 2018).

In some cases, the evaluations on topical products which boast effectiveness, such as anti-inflammatory activity, require a preliminary induction of a controlled erythema condition. In these cases, the use of the reflectance spectroscopy technique is essential both to monitor the establishment of a suitable degree of chemically induced inflammation of the skin, in terms of erythema and to monitor the reduction of redness thanks to the anti-inflammatory activity of the formulation.

This is a typical approach used by our research group to evaluate the anti-inflammatory efficacy of a new topical formulation (Cristiano et al., 2022; Cristiano et al., 2021; Barone et al., 2020; Bruno et al., 2022; Rinaldi et al., 2022; d Avanzo et al., 2022). The protocol provides for the application of an aqueous solution of methyl-nicotinate (0.2% w/v) for the chemical induction of erythema. Methyl nicotinate is a rubefacient substance that induces local erythema and redness, due to a transient increase in the skin perfusion (Elawa et al., 2020). It is important to consider that the erythematous effect of methyl nicotinate is concentration- and contact time-dependent, and reflectance spectroscopy can be employed to monitor the change in skin color and to measure differences in cutaneous inflammatory reaction between the control (placebo)-treated group and the formulation-treated group (Jumbelic et al., 2006). Thanks to this investigation approach, it is possible to evaluate the efficacy of a new formulation or new ingredient in terms of inflammation signs reduction using the non-invasive but objective technique, also safeguarding the wellbeing of the subject. In fact, given the high sensitivity of reflectance spectroscopy, it is eventually possible to stop the *in vivo* experimentation when the redness exceeds certain limits, even those not visible to the naked eye.

This is particularly useful when the erythema is physically inducted, as in the case of UV lamp irradiation. UVB-induced skin erythema can be monitored, again using reflectance spectroscopy, when the effectiveness of topical photoprotective products must be investigated. In a scientific work carried out by Saija et al. (2000), reflectance spectroscopy was used to evaluate the efficacy of caffeic and ferulic acids as topical photoprotective molecules. The authors induced skin erythema on human volunteers by using an ultraviolet lamp, emitting in the range 290–320 nm, and the induction was monitored using a reflectance visible spectrophotometer X-Rite. After obtaining a suitable but not noxious erythema, the inflamed skin sites of human volunteers were treated with a saturated aqueous solution of caffeic or ferulic acid. At the end of the time foreseen for the treatments, any reduction of photoinduced erythema and recovery of physiological parameters were investigated through reflectance spectroscopy comparing results obtained by sites treated with caffeic and ferulic acids with that of untreated sites. These two hydroxycinnamic acids proved to afford relevant cutaneous protection against UV-B-induced erythema, correlated to their antioxidant/radical scavenging effectiveness. Indeed a great reduction in erythema index values was recorded over time on treated skin sites previously exposed to UV-B radiation and this reduction was relevant if compared to the physiological recovery obtained in untreated sites.

Reflectance spectroscopy is not only important in evaluating the effects induced by cosmetic and pharmaceutical products for topical use on the skin but it also plays a fundamental role in the characterization of cutaneous formulations. Such an example reflectance spectroscopy is involved in the detection of any unauthorized whitening substances that could cause damage to the skin for chronic treatments as well as being able to detect substances present in lipsticks and nail polishes, playing a fundamental role in forensic purposes (Deconinck et al., 2014; Wong et al., 2019; Chophi et al., 2021).

## 4 Conclusion and future perspectives

In conclusion, reflectance spectroscopy emerges as a valuable tool for investigating several dermatological conditions and evaluating skin reactions to topical products in a non-invasive, objective, and quantitative manner. By harnessing the power of light, this technique offers unprecedented insights into the complex interplay between skin and topical interventions, paving the way for enhanced product development and personalized skincare regimens. Indeed, its ability to provide qualitative and quantitative information about cutaneous features, such as pigmentation and microcirculation offers valuable insight for both clinical diagnosis and product formulation. Its non-destructive assets make it particularly suitable for longitudinal studies and daily monitoring of treatment progress. However, further research is needed to standardize protocols, improve data interpretations, and validate its efficacy across different formulations and skin types. The standardization of spectroscopic investigation on human skin is fundamental to reduce errors. A better clarification of the skin components responsible for the optical properties of the skin and the resulting spectroscopic signals can widen the scope of applicability of reflectance spectroscopy in the dermatological field and be even more reliable in predicting the onset of skin pathologies. Nevertheless, considering the constant advancement in technology, reflectance spectroscopy holds great potential in influencing the field of dermatology and cosmetology, contributing to the improvement of patient care and product safety.
